# Supplementary material for: The Extract of Gloiopeltis tenax Enhances Myogenesis and Alleviates Dexamethasone-Induced Muscle Atrophy
Source: Int J Mol Sci. 2024 Jun 20;25(12):6806. doi: 10.3390/ijms25126806 (PMC11203874; doi:10.3390/ijms25126806)
Supplement: Supplementary file 1 [file ijms-25-06806-s001.zip › ijms-3018407-supplementary.pdf]

Supplementary Materials

The extract of *Gloiopeltis tenax* enhances myogenesis and alleviates dexamethasone-induced muscle atrophy

Si-Hyung Kim[1,†], Young-Eun Leem[1,†], Hye Eun Park[2], Hae-In Jeong[2], Jihye Lee[2,*] and Jong-Sun Kang[1,*]

[1] Department of Molecular Cell Biology, Sungkyunkwan University, School of Medicine, Suwon, Republic of Korea; shk990929@skku.edu (S.-H.K.); leemyo@skku.edu (Y.-E.L.); Kangj01@skku.edu (J.-S.K.)

[2] Laboratories of Marine New Drugs, REDONE TECHNOLOGIES CO.,LTD, Jeollanamdo 57247, Republic of Korea; phe@urc.kr (H.E.P); jhi@urc.kr (H.-I.J.); ljh@urc.kr (J.L.)

* Correspondence: ljh@urc.kr (J.L.); Kangj01@skku.edu (J.-S.K.); Tel.: +82-70-4219-1632 (J.L.); +82-30-299-6135 (J.-S.K.)

Table of Contents

Figure S1. LC-MS spectrum of GTAE **S3**

Figure S2. HPLC spectrum of GTAE **S4**

Figure S3. ^{1}H NMR spectrum (400 MHz, D_2O) of 4-hydroxy-*N*-methylproline **S5**

Figure S4. ^{1}H NMR spectrum (400 MHz, D_2O) of floridoside **S5**

Figure S5. ^{1}H NMR spectrum (400 MHz, CD_3OD) of shinorine **S6**

Figure S6. The raw data images of the Western blot shown in Figure 1h **S7**

Figure S7. The raw data images of the Western blot shown in Figure 1j **S8**

Figure S8. The raw data images of the Western blot shown in Figure 2b **S9**

Figure S9. The raw data images of the Western blot shown in Figure 3f **S10**

Figure S10. The raw data image of the Western blot shown in Figure 5b **S11**

Table S1. The primary antibodies used in this study **S12**

Table S2. The primer sequence for qRT-PCR **S13**

Figure S1. LC-MS spectrum of GTAE

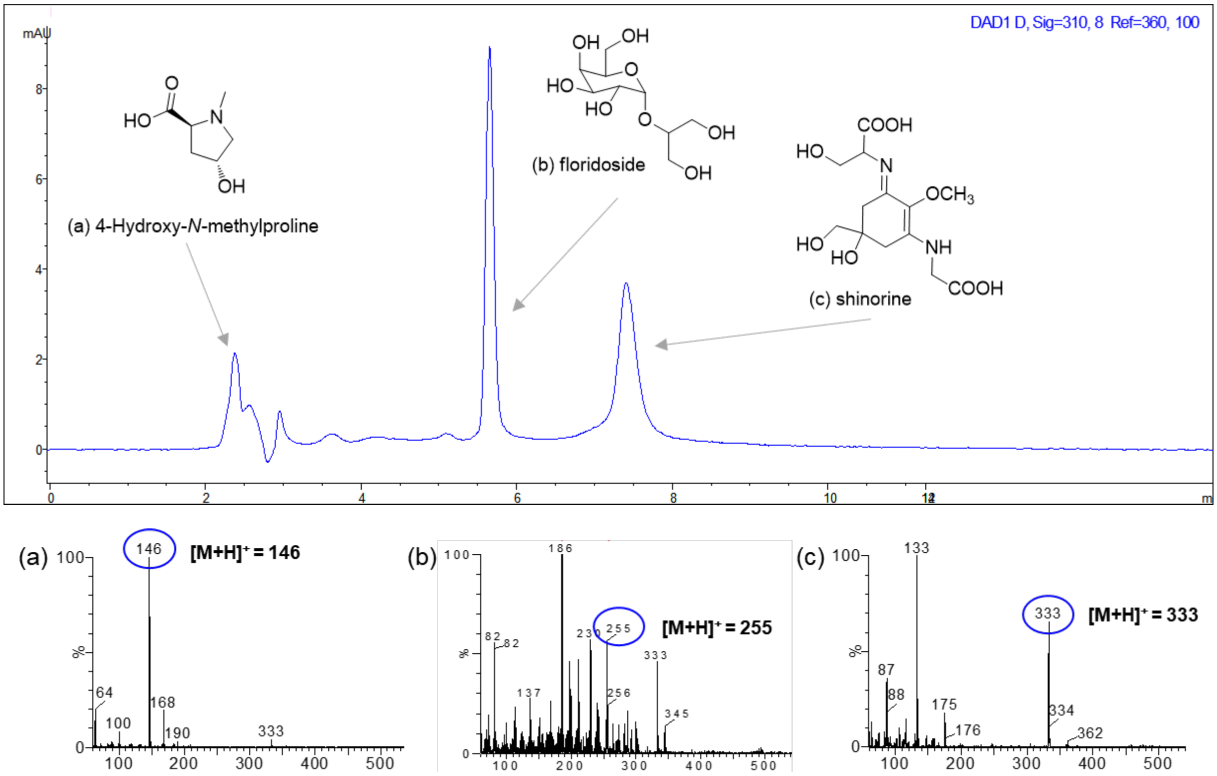

Figure S2. HPLC spectrum of GTAE

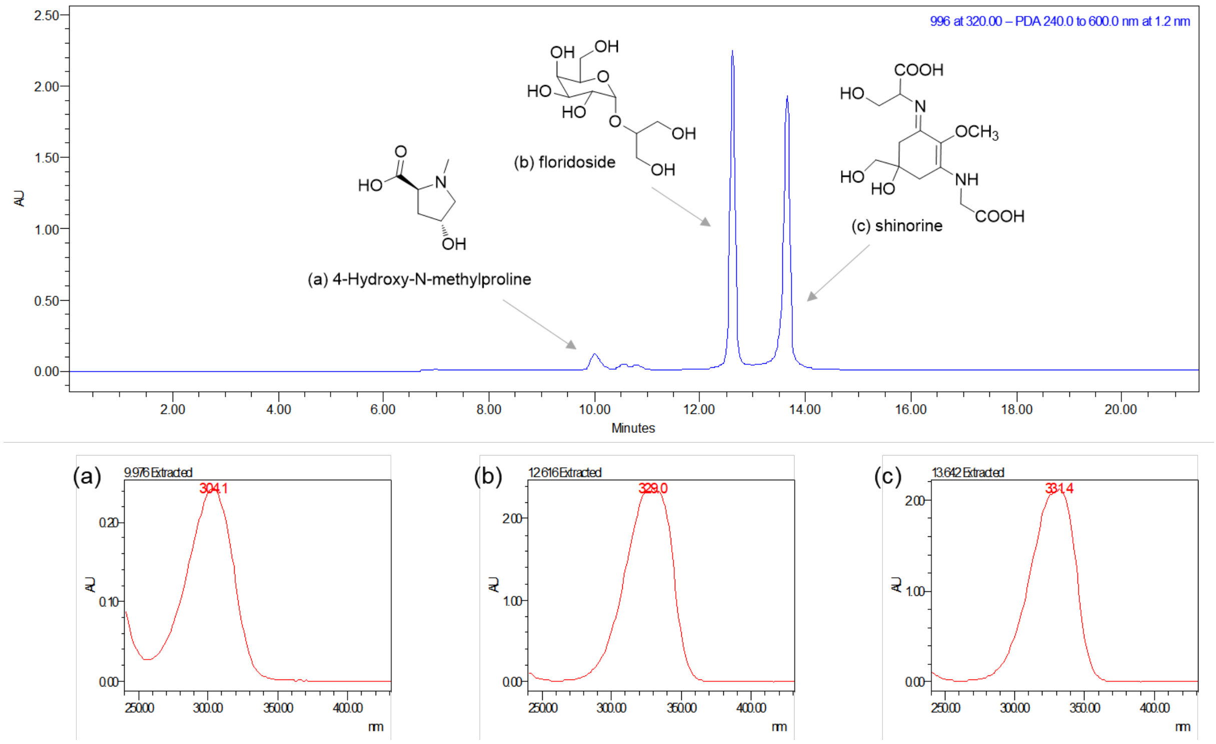

Figure S3. ^{1}H NMR spectrum (400 MHz, D_2O) of 4-hydroxy-*N*-methylproline

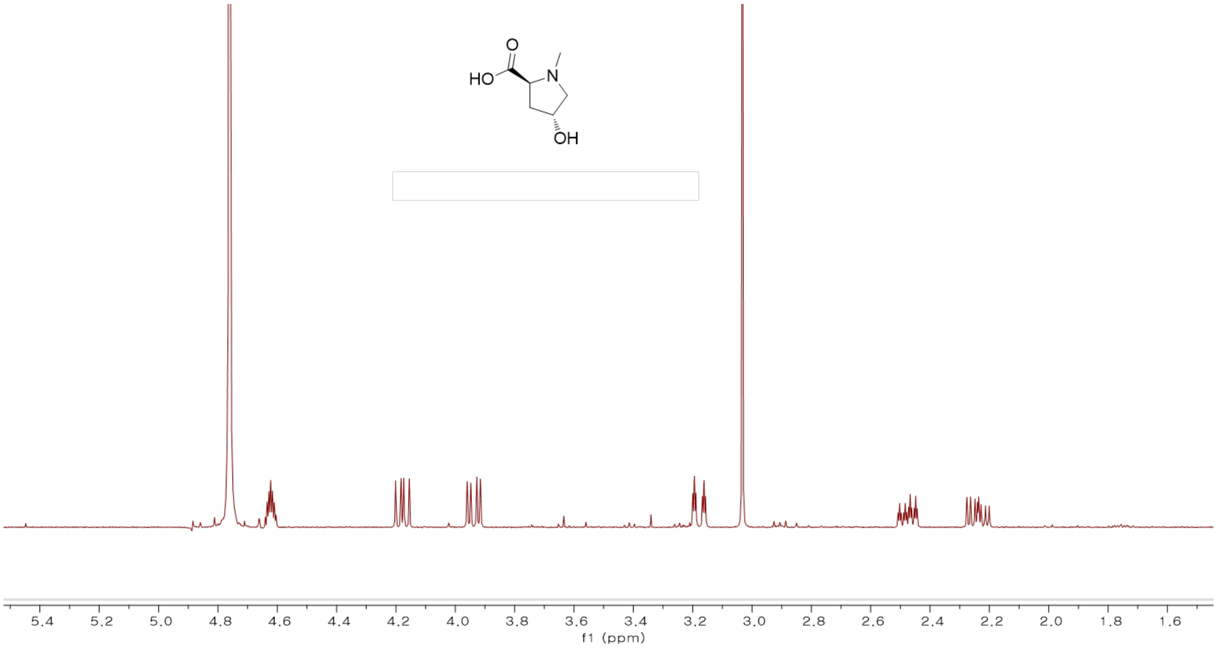

Figure S4. ^{1}H NMR spectrum (400 MHz, D_2O) of floridoside

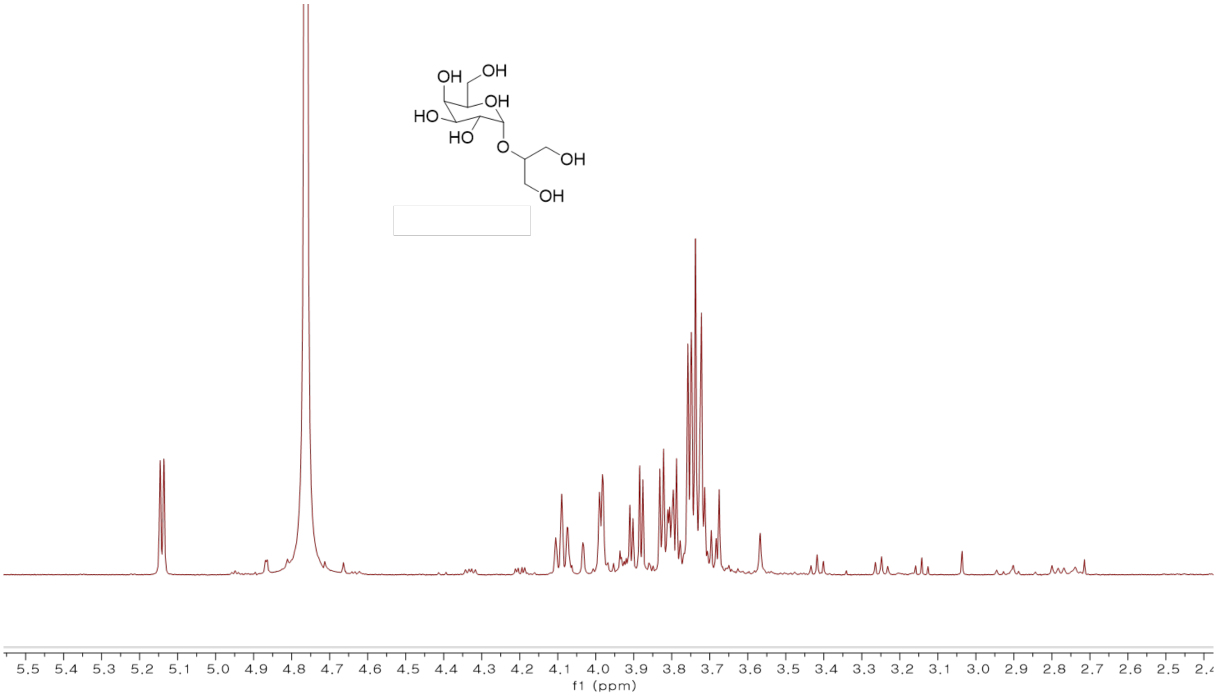

Figure S5. ^{1}H NMR spectrum (400 MHz, CD_3OD) of shinorine

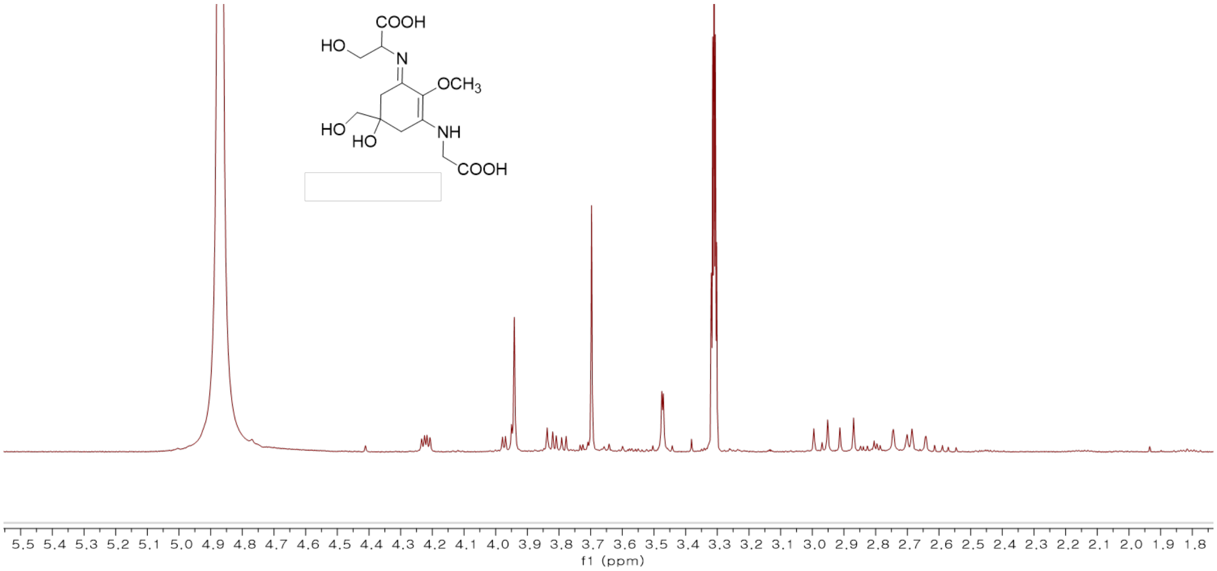

Figure S6. The raw data images of the Western blot shown in Figure 1h

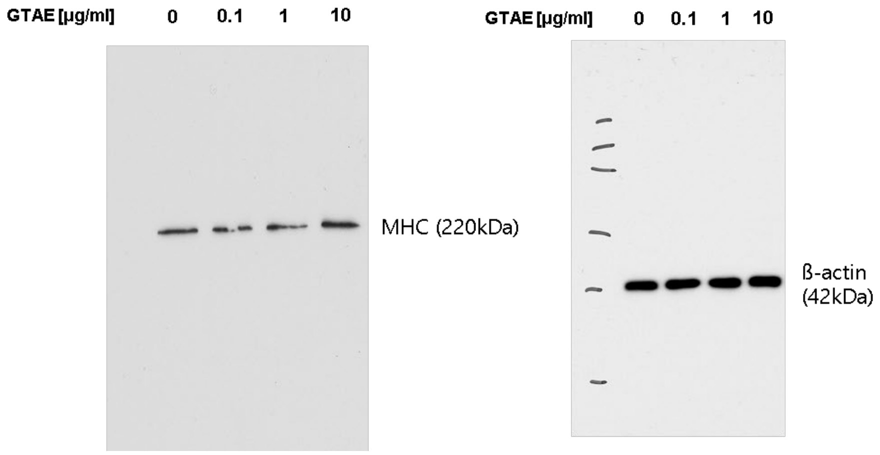

Figure S7. The raw data images of the Western blot shown in Figure 1j

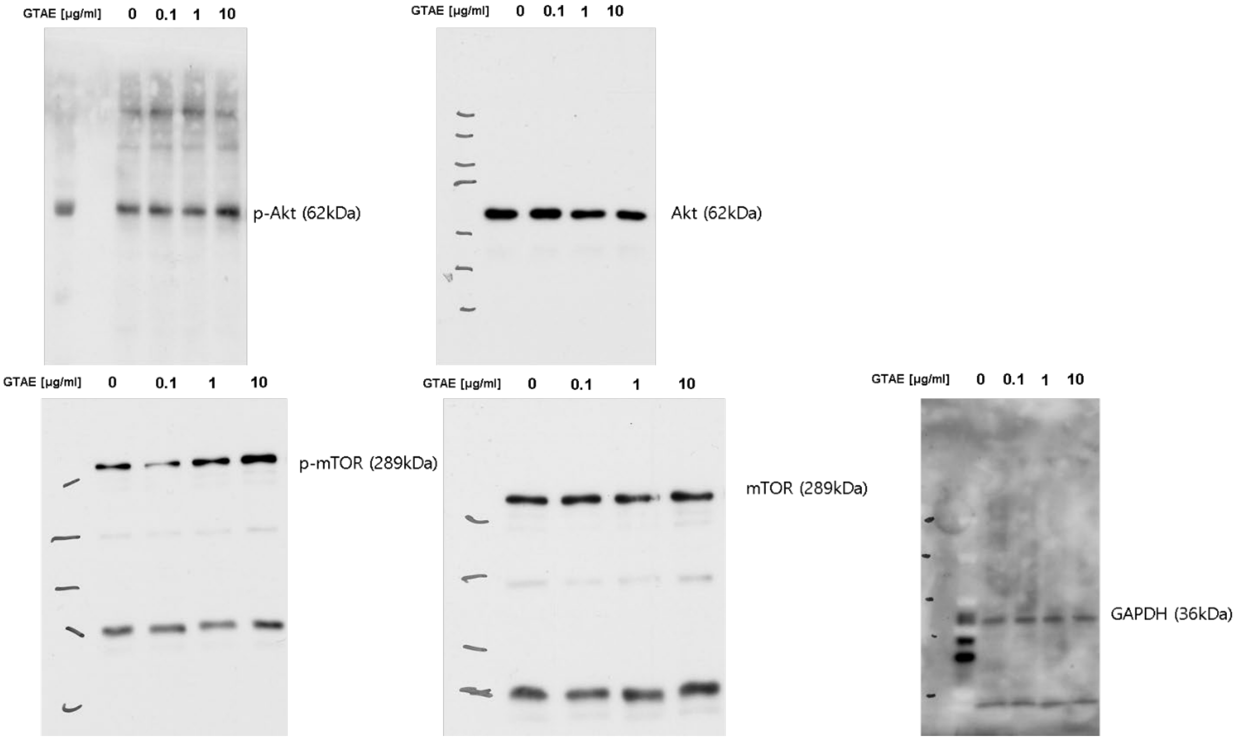

Figure S8. The raw data images of the Western blot shown in Figure 2b

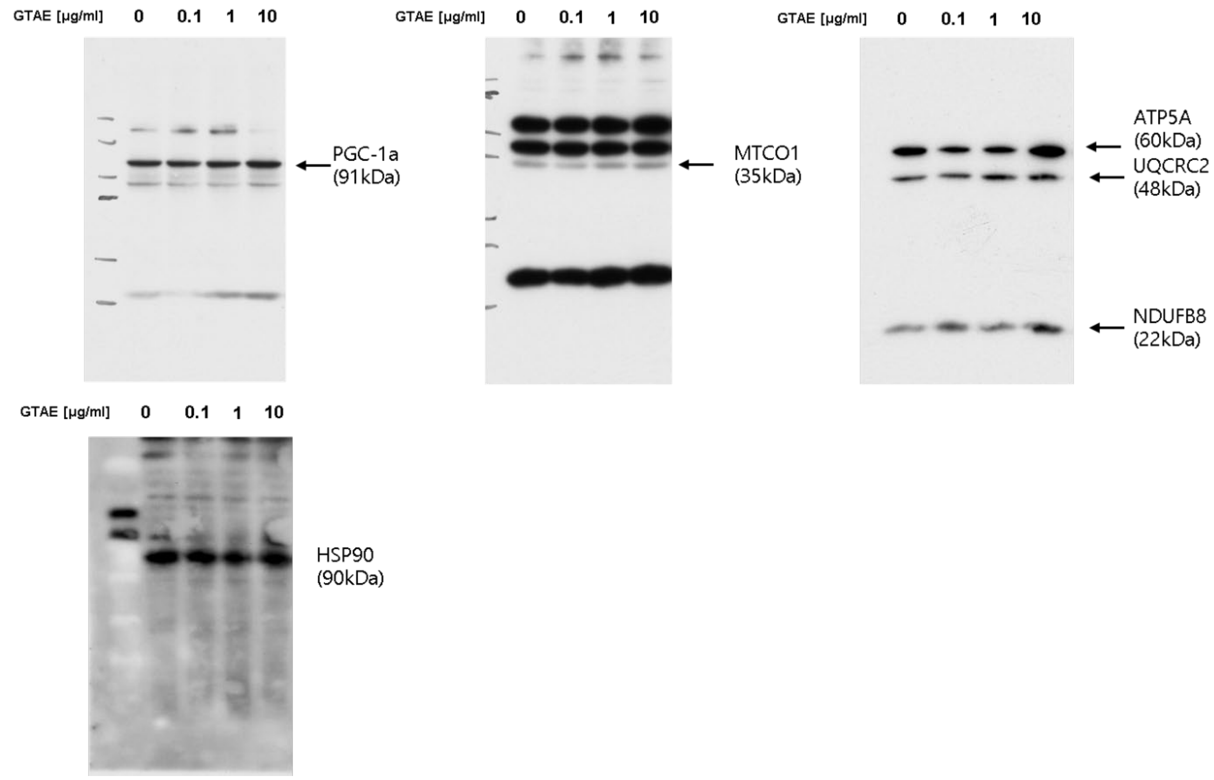

Figure S9. The raw data images of the Western blot shown in Figure 3f

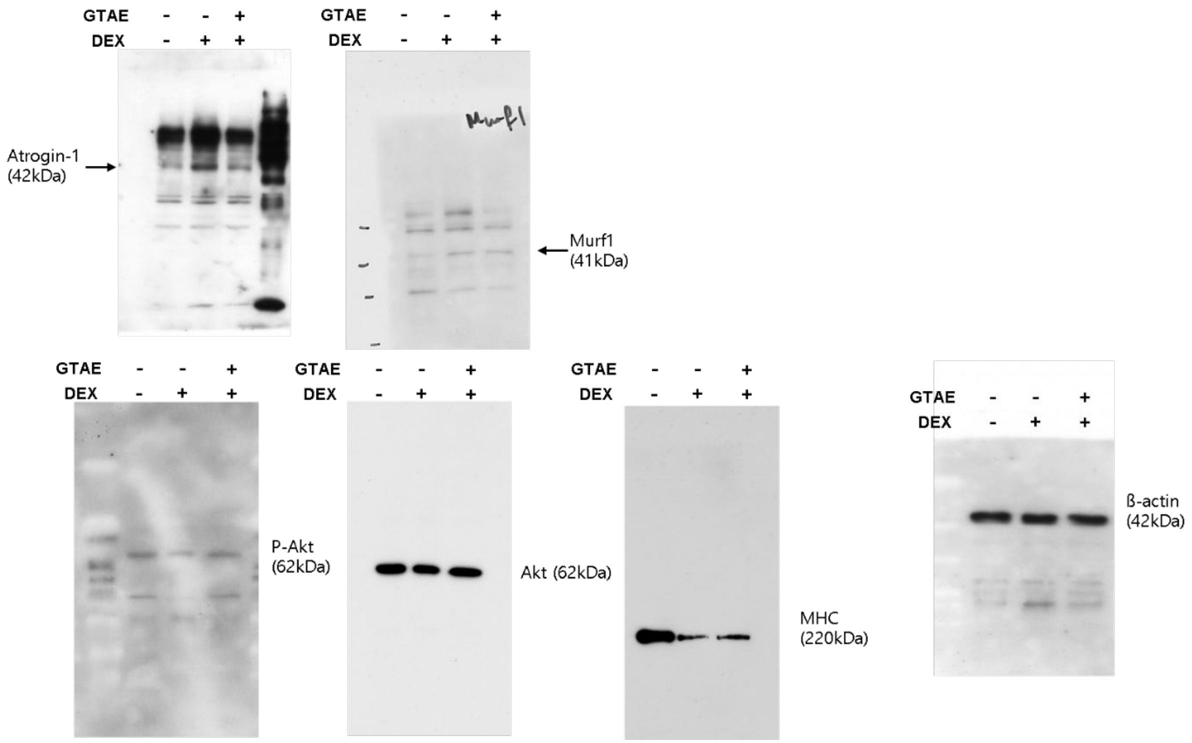

Figure S10. The raw data image of the Western blot shown in Figure 5b

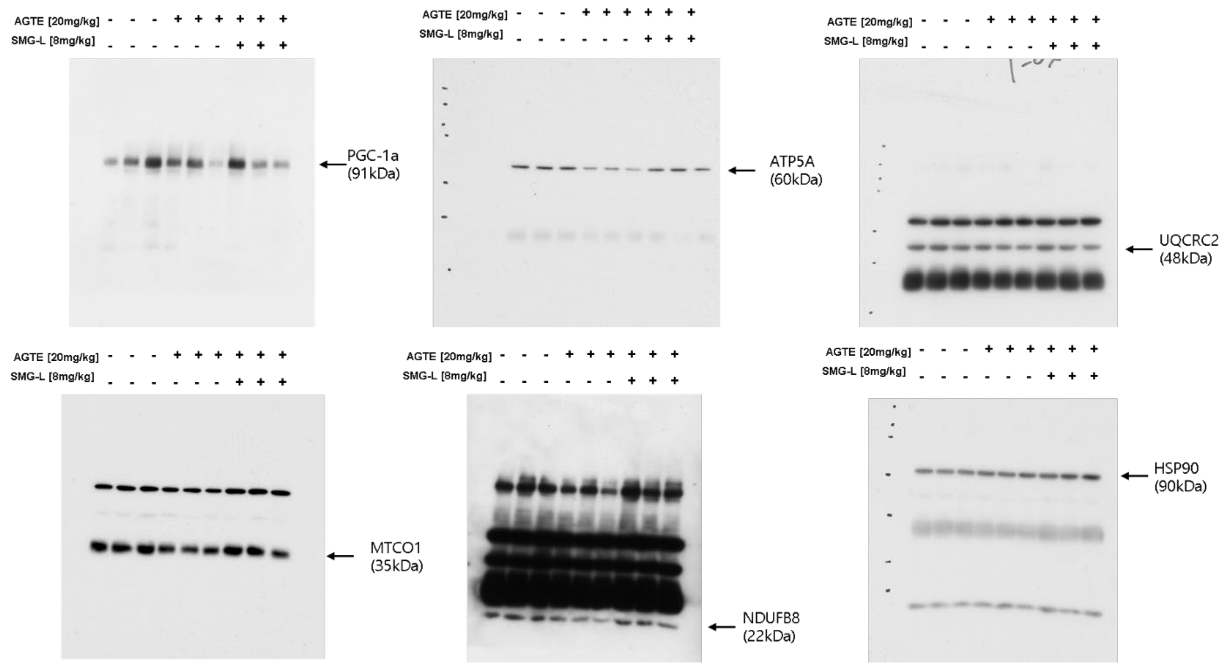

Table S1. The primary antibodies used in this study

Antigen	Cat. No.	Manufacturer
**MHC**	MF 20	Developmental Studies Hybridoma Bank (DSHB)
**Total-OxPHOS**	Ab110413	Abcam
**Akt**	9272	Cell Signaling
**p-Akt**	9275	Cell Signaling
**p-mTOR**	5536	Cell Signaling
**mTOR**	2972	Cell Signaling
**MuRF1**	PA5-76695	Invitrogen
**Atrogin-1**	Sc-33782	Santa Cruz
**PGC-1α**	ST1202-1SETCN	Calbiochem
**HSP90**	Ab13495	Abcam
**β-Actin**	4970	Sigma-Aldrich
**GAPDH**	LF-PA0212	Abfrontier

Table S2. The primer sequence for qRT-PCR

**Gene**		**Sequences**
Tfam	Forward	5'-CTGATGGCCATTACATGT-3'
	Reverse	5'-AAAGCCCGGAAGGTTAG-3'
Atp5a	Forward	5'-CGATCTATCCAAGCAGGCTGT-3'
	Reverse	5'-GCCACCCACCAAAGGAATCGT-3'
UQCRC	Forward	5’-GCACTTACGGTGGTGGAGTG-3’
	Reverse	5’-GGTACATAGGCGCATCCACTG-3’
MTCO	Forward	5’-CTACTATTCGGAGCCTGAGC-3’
	Reverse	5’-GCATGGGCAGTTACGATAAC-3’
SDHB	Forward	5’-ACCCCTTCTCTGTCTACCG-3’
	Reverse	5’-AATGCTCGCTTCTCCTTGTAG-3’
UCP2	Forward	5'-ACTGTCGAAGCCTACAAGAC-3'
	Reverse	5'-CACCAGCTCAGTACAGTTGA-3'
PGC-1α	Forward	5'-ATGTGTCGCCTTCTTGCTCT-3'
	Reverse	5'-CGGTGTCTGTAGTGGCTTGA-3'
Atrogin-1	Forward	5’-CAACATTAACATGTGGGTGTAT-3’
	Reverse	5’-GTCACTCAGCCTCTGCATG-3’
Murf1	Forward	5’-GAGAACCTGGAGAAGCAGCT-3’
	Reverse	5’-CCGCGGTTGGTCCAGTAG-3’
eMHC(Myh3)	Forward	5'-CTTCACCTCTAGCCGGATGGT-3'
	Reverse	5'-AATTGTCAGGAGCCACGAAAAT-3'
Myogenin	Forward	5'-ATCTCCGCTACAGAGGCGGG-3'
	Reverse	5'-TAGGGTCAGGCCGCGAGCAAA-3'
Mt-co1	Forward	5'-CTACTATTCGGAGCCTGAGC-3'
	Reverse	5'-GCATGGGCAGTTACGATAAC-3'
β-Actin	Forward	5’-CATTGCTGACAGGATGCAGAAGG-3’
	Reverse	5’-TGCTGGAAGGTGGACAGTGAGG-3’
L32	Forward	5'-GGCCTCTGGTGAAGCCCAAGATCG-3'
	Reverse	5'-CCTCTGGGTTTCCGCCAGTTTCGC-3'
